# Thinking About It All Together: A Descriptive Analysis to Understand Comorbidities in People Living With Dementia

**DOI:** 10.1002/hsr2.70449

**Published:** 2025-02-05

**Authors:** Yuchen Zhang, Zhirui Deng, Jennifer Seaman, Theresa A. Koleck

**Affiliations:** ^1^ Department of Acute & Tertiary Care University of Pittsburgh, School of Nursing Pittsburgh Pennsylvania USA; ^2^ Office of Research and Scholarship University of Pittsburgh, School of Nursing Pittsburgh Pennsylvania USA; ^3^ Department of Health Promotion & Development University of Pittsburgh School of Nursing Pittsburgh Pennsylvania USA

**Keywords:** Alzheimer's disease and related dementia, comorbid conditions, comorbidity profile, dementia care, person‐centered care

## Abstract

**Background and Aims:**

The prevalence of Alzheimer's disease and related dementia (ADRD) is on the rise. There is a corresponding escalation in the number of persons living with dementia who require complex, longitudinal support in care due to the progressive declines in cognitive and clinical profiles of persons living with dementia when delivering individualized person‐centered care that promotes overall health and well‐being. Hence, we aim to describe the presence and patterns of co‐occurring comorbidities in persons living with dementia.

**Methods:**

This study is a retrospective, cross‐sectional descriptive analysis based on curated electronic health record data from the *All of Us* Research Program from October 1, 2015, to June 30, 2022. We included individuals who were 65 years of age or older with at least one dementia‐related diagnosis. We categorized 14 comorbidities by the Charlson Comorbidity Index, and defined all diseases based on the International Classification of Diseases Tenth Revision Diagnosis codes. We employed descriptive statistics and visualized data with UpSet Plots. Demographic characteristics (i.e., age, sex, race, and ethnicity) between people with and without co‐occurring comorbidities were compared with either chi‐square or Wilcoxon signed‐rank tests.

**Results:**

Persons living with dementia (*N* = 4003) were a mean of 73 years old, 72.5% non‐Hispanic White, and 47.5% female. Approximately 87% of persons living with dementia were diagnosed with at least one additional comorbidity. The most common comorbidities included diabetes (67.82%), renal disease (40.24%), chronic pulmonary disease (39.85%), congestive heart failure (37.37%), and peripheral vascular disease (34.57%). Heterogeneous patterns of co‐occurring comorbidity were noted among persons living with dementia.

**Conclusion:**

Our study demonstrates the high prevalence of co‐occurring comorbid illness among persons living with dementia. It is critical that the impact of these co‐occurring conditions on patients' disease trajectories be better understood to promote treatment choices that are person‐centered and goal‐concordant.

## Summary

- Comorbid conditions are highly prevalent in persons living with dementia and heterogeneous patterns of co‐occurring conditions exist.
- Heterogenous comorbidity profiles in persons living with dementia illustrate the complex health challenges faced by this vulnerable population.
- The systematically characterized prevalence and patterns of co‐occurring comorbidities in persons living with dementia enhance our understanding of the multifaceted needs of persons living with dementia.

## 1 Background

By 2060, up to 14 million Americans who are 65 years of age and older will be living with Alzheimer's disease [1]. Additionally, around 70%–90% of persons living with dementia will have at least one comorbid condition, posing additional needs for health‐related care and management [2]. The projected total annual cost of dementia will exceed $1.8 trillion by 2050 [3], and this expense will only increase without the introduction of more efficient models of care. Dementia‐related syndromes are terminal conditions requiring complex longitudinal care management due to progressive worsening of disease‐related symptoms with functional declines [2, 4, 5]. Those declines foreshadow persons living with dementia experiencing increased difficulty in communicating and managing chronic conditions, as well as in engaging in daily health maintenance activities, leading to escalated risks of underdiagnosis, overtreatment, and negative health outcomes [5, 6, 7, 8]. The paradoxical situation underlines the critical importance of acquiring an accurate understanding of the disease profile for a person living with dementia to provide appropriate person‐centered, comprehensive care support along with effective disease management.

Focusing on the overall health status of persons living with dementia, rather than specific diseases, allows for a more holistic approach in person‐centered care [4, 9, 10]. The coexistence of dementia and comorbidities complicates the health profile and care‐related needs in persons living with dementia. Especially when experiencing cognitive deterioration, persons living with dementia encounter vulnerability and increased risk of receiving potentially inappropriate care that compromises their quality of life and dignity, accelerates functional and cognitive declines, exacerbated distressing symptoms, amplifies care‐related burdens, and compromises their general quality of life [8, 11, 12, 13, 14, 15]. The presence of dementia is also likely to heighten the likelihood for persons living with dementia to encounter deficiencies in quality of care [5, 7, 8]. A better understanding of the co‐occurring chronic conditions in persons living with dementia would allow the development of comprehensive care to support personalized care planning and informed decision‐making based on individuals' personal values and wishes, while reducing care‐related costs and burdens and enhancing the well‐being of persons living with dementia.

While comorbidities commonly exist in persons living with dementia and are frequently associated with a burdensome clinical profile [5, 8], the heterogeneity of those coexisting chronic conditions has yet to be characterized. The *All of Us* (AoU) Research Program is a continuous effort to gather health‐related information from more than one million people living in the United States and make this data broadly available to researchers [16]. A population‐based approach offers the ability to evaluate the prevalence of multiple diseases in a specific population [17], and AoU provides a unique opportunity to comprehensively describe the prevalence of co‐occurring chronic conditions in persons living with dementia. For this study, we explored the presence of comorbidities and patterns of co‐occurring chronic conditions in persons living with dementia enrolled in AoU‐based electronic health record (EHR) condition diagnosis codes.

## 2 Methods

### 2.1 Sample and Setting

#### 2.1.1 AoU Research Program

AoU (allofus.nih.gov) is a large‐scale, nationwide initiative in the United States led by the National Institutes of Health aiming to gather health data and accelerate research to improve population health by enabling individualized treatment and care [16]. Participant recruitment was conducted through participating health organizations and Federally Qualified Health Centers. Initial activities of this program, including study enrollment, completing informed consent, agreeing to share EHRs, and administering baseline health surveys, are conducted digitally through the AoU website. As of July 2024, about 563,900 participants have completed the initial steps of the program [18]. Approved researchers who complete the training modules are granted access to individual‐level data with various tiers of restrictions.

### 2.2 Cohort Identification

Our team (first, second, and senior author) completed all required steps, including confirming the institutional Data Use and Registration Agreement, verifying identities, completing responsible and ethical research trainings, and signing the Data User Code of Conduct) to access deidentified individual‐level data through the AoU Researcher Workbench. Using the AoU Researcher Workbench Controlled Tier Data set version 7, we identified a cohort of older adults (65 years of age and older) with available EHR data containing at least one dementia‐related International Classification of Diseases Tenth Revision (ICD‐10) diagnosis code (Supplemental Material 1) between when ICD‐10 codes were clinically implemented (October 1, 2015) to the latest data available in AoU (June 30, 2022) [19]. We calculated participants' ages based on their dates of birth and the date of the implementation of ICD‐10 diagnosis codes (October 1, 2015). We curated the data set with participants' demographic information and comorbidities based on the defined ICD‐10 diagnostic codes (Supplementary Material 1).

### 2.3 Measures

The demographics of the participants (i.e., age, sex, race, and ethnicity) were based on the self‐reported information provided upon study enrollment. Comorbidities (*n* = 14); that is, diabetes with and without complications, mild and severe renal disease, chronic pulmonary disease, congestive heart failure, peripheral vascular disease, cerebrovascular accident, malignancy, myocardial infarction, mild and severe liver disease, rheumatoid arthritis, metastatic solid tumor, hemiplegia, peptic ulcer, and HIV/AIDS) were operationalized based on the defined categories in Charlson Comorbidity Index (CCI), which has been recognized as the gold‐standard measure to evaluate comorbidity in clinical research [20]. Presence of a comorbid condition was determined based on documentation of one or more relevant ICD‐10 codes between October 1, 2015, and June 30, 2022. Relevant ICD‐10 diagnostic codes were identified through a combination of previous literature [21, 22] and the Chronic Conditions Data Warehouse developed by the Centers for Medicare and Medicaid Services [23]. Defined ICD‐10 diagnosis codes for each condition are listed in Supplementary Material 1. We, further, obtained participants' visit occurrence related to relevant ICD‐10 codes to describe the number of healthcare visits or encounters.

### 2.4 Statistical Analysis

We conducted a cross‐sectional, retrospective, descriptive analysis through the AoU Researcher Workbench using R programming language (version 4.4.0) in a Jupiter notebook environment. We calculated descriptive statistics for all variables, including means, medians, standard deviations, interquartile ranges, percentages, and counts, as appropriate. We visualized data using UpSet plots (UpSetR package) and explored the patterns of mutually co‐occurring comorbidities in persons living with dementia [24]. Disease categories in CCI representing various severities of the disease (e.g., diabetes with complications and diabetes without complications) were grouped into a single condition to enhance clinical meaningfulness of the resulting plots. Any variable with a count of 20 or fewer was either omitted from the report, combined with other relevant output variables, or removed from graphs to maintain participants' privacy. [16] We compared age and dichotomized sex (male vs. female), race (White vs. underrepresented individuals in biomedical research), and ethnicity (Hispanic or Latino vs. non‐Hispanic or Latino) between participants with dementia only and participants with dementia and other comorbidities using chi‐square or two‐tailed Wilcoxon signed‐rank tests as appropriate. A *p*‐value of < 0.05 was considered statistically significant.

## 3 Results

Participants (*N* = 4003) were on average, 73 years of age, 47.5% female, and 72.5% non‐Hispanic White. The median number of visits for dementia and comorbidities was 63 per participant with approximately 70.8% related to outpatient or primary care. About 87% (*n* = 3488) of participants had at least one comorbid condition documented while living with dementia. The top five most common comorbid conditions were diabetes (67.82%), renal disease (40.24%), chronic pulmonary disease (39.85%), congestive heart failure (37.37%), and peripheral vascular disease (34.57%). HIV/AIDS (0.97%) was the least common disease. Table 1 presents additional information about sample characteristics and the disease profile of the selected cohort.

**Table 1:** Demographic characteristics and disease profile of participants with dementia (*N* = 4003).

Variable	Statistic
Age (mean, SD)	73.01 (5.93)
Sex (*n*, %)	
Female	1900 (47.5)
Male	1993 (49.8)
Others/Missing	112 (2.80)
Race/ethnicity (*n*, %)	
Asian/non‐Hispanic	43 (1.74)
Black/non‐Hispanic	399 (9.97)
White/non‐Hispanic	2904 (72.55)
Other/non‐Hispanic	56 (1.40)
Hispanic/any race	424 (10.59)
Missing	182 (4.55)
Number of visits (median, interquartile range)	63 (135)
Setting of each visit occurrence (*n*, %)	
Inpatient setting	91,018 (16.98)
Outpatient setting	379,745 (70.83)
Missing	65,398 (12.20)
Numbers of comorbid conditions participant diagnosed with (mean, SD)	4.32 (2.50)
Total numbers of comorbid conditions participant diagnosed with (*n*, %)	
1 (having dementia only)	515 (12.87)
2	624 (15.59)
3	588 (14.69)
4	578 (14.44)
5	497 (12.42)
6	408 (10.19)
7	287 (7.17)
8	221 (5.52)
9	154 (3.85)
10 or more	131 (3.27)
Comorbid condition prevalence (*n*, %)	
Diabetes	2715 (67.82)
Without complications	1581 (39.50)
With complications	1134 (28.33)
Renal disease	1611 (40.24)
Mild to moderate	1338 (33.42)
Severe	273 (6.82)
Chronic pulmonary disease	1595 (39.85)
Congestive heart failure	1496 (37.37)
Peripheral vascular disease	1384 (34.57)
Cerebrovascular accident	1286 (32.13)
Malignancy	797 (19.91)
Myocardial infarction	792 (19.79)
Liver disease	598 (13.94)
Mild to moderate	500 (12.49)
Severe	98 (2.45)
Rheumatoid arthritis	359 (8.97)
Metastatic solid tumor	273 (6.82)
Hemiplegia	239 (5.97)
Peptic ulcer	124 (3.10)
HIV/AIDS	39 (0.97)

**Figure 1:**
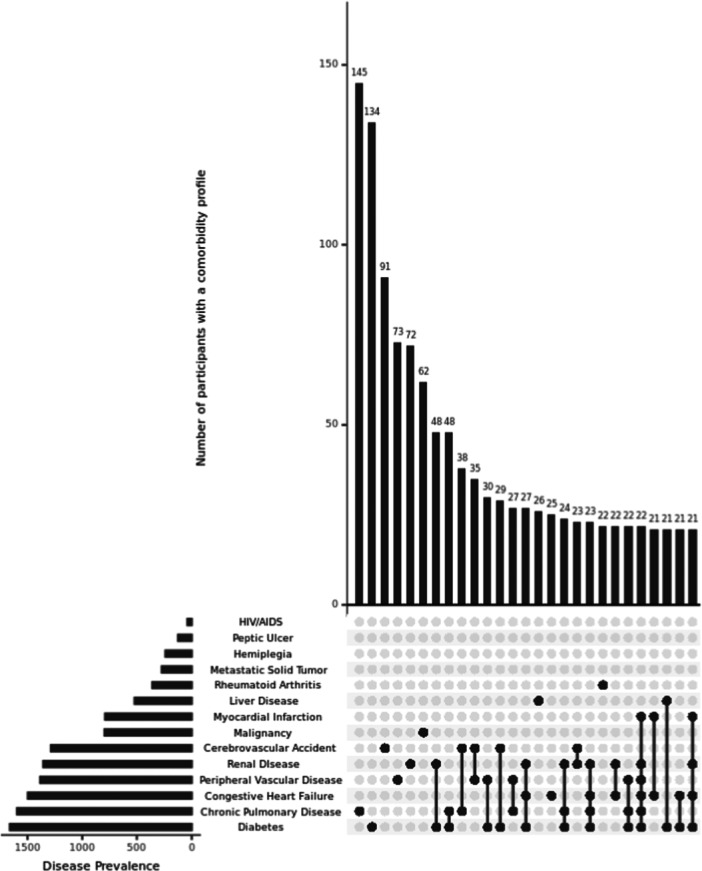
Patterns of comorbidity profiles in people living with dementia (*N* = 4003) with counts of > 20 people. *Note:* Disease categories in Charlson Comorbidity Index representing various severities of the disease were grouped into a single condition.

**Figure 2:**
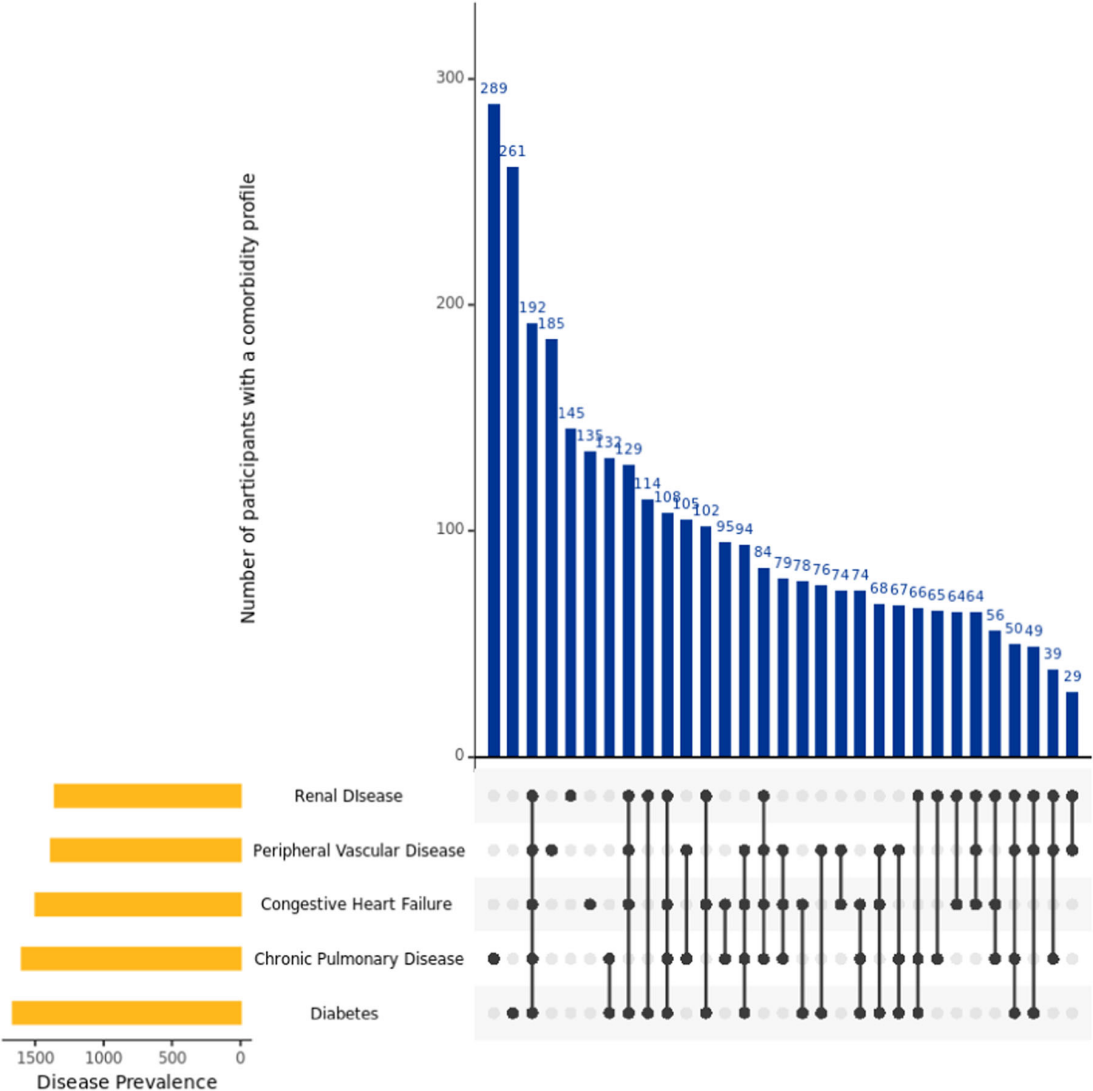
Patterns of comorbidity profiles of the five most prevalent comorbid diseases in people living with dementia with counts of > 20 participants. *Note:* Disease categories in Charlson Comorbidity Index representing various severities of the disease were grouped into a single condition.

Figure 1 displays the co‐occurring comorbidity patterns of all included comorbidities, and Figure 2 presents the co‐occurring patterns of the top five most prevalent comorbidities in the study sample. The intersection size of overlapping patterns of comorbidities increased as the analysis focused on the more prevalent comorbid conditions with about 5% of sample (*n* = 192) diagnosed with all five comorbid conditions, and 10.37% of the sample (*n* = 415) diagnosed with various combinations of four out of five comorbidities (Figure 2). Overall, both graphs demonstrated a heterogenous distribution of comorbidity patterns in persons living with dementia. Last, compared to people diagnosed with dementia only, the presence of comorbidities in persons living with dementia was associated with being female (*χ*² = 14.31, *df* = 1, *p* < 0.001) and identifying as a race other than White (*χ*² = 15.11, *df* = 1, *p* < 0.001), but not associated with age (*W* = 922869, *p* = 0.31) or ethnicity (*χ*² = 14.31, *df* = 1, *p* = 0.054).

## 4 Discussion

Our study highlights the heterogenous patterns of comorbid conditions in persons living with dementia, and the ubiquitous presence of comorbidities in persons living with dementia at a population level. While only a small minority (12.9%) had no comorbidities, average persons living with dementia had about four co‐occurring chronic conditions. In addition, focusing the analysis on the most prevalent comorbidities in persons living with dementia revealed distinct, and potentially more clinically meaningful, co‐occurring comorbidity patterns. These exploratory study findings indicate the need to further unfold the complex relationship of comorbidities in persons living with dementia when considering how to develop comprehensive person‐centered care with tailored, targeted individualized approaches for persons living with dementia.

Comorbidities have consistently been a crucial consideration in research related to Alzheimer's disease and related dementias (ADRD). Our analysis highlighted the widespread presence of co‐occurring comorbid conditions among persons living with dementia in the United States. Descriptive studies from other countries, such as the United Kingdom, have reported similar results [25]. Previous research has also recognized that the presence of additional health conditions can significantly influence disease diagnosis and cognitive declines. Specifically, comorbidity patterns are associated with dementia subtypes and different clinical profiles, which are further correlated with the biomechanics and rate of ADRD‐related disease progression and cognitive trajectories [26, 27, 28]. However, there is a notable gap in research to address the full spectrum of health challenges faced by persons living with dementia with overall care strategies that include both dementia‐related syndromes and distressing symptoms from other chronic conditions [5, 26, 27]. Because additional health conditions complicate symptom management, frequent decision‐making for care that aligns with treatment preferences is also needed to optimize overall quality of life in persons living with dementia [2, 5, 29, 30]. Other than those symptom burdens, persons living with dementia also face a higher risk of polypharmacy due to comorbid physical diseases, which are both associated with worsening of cognition, functional ability, and overall survival in persons living with dementia [8, 15].

With the initiation of the Guiding an Improved Dementia Experience (GUIDE) model by the Centers for Medicare & Medicaid Services for the purpose of high‐quality, coordinated care in persons living with dementia [31], future studies should consider the impact of heterogenous comorbidity profiles on dementia care when developing interventions. Moreover, additional clinical factors in persons living with dementia should be incorporated in care models and clinical care guidelines to fulfill the multifaceted needs in dementia care [2, 4, 5]. Our analysis demonstrated the existence of frequent co‐occurring comorbidity profiles in persons living with dementia. Clinical subgroups could be further developed in future studies for efficient, tailored interventions that promote the overall quality of life in persons living with dementia.

Limitations exist in this study. First, while the AoU study attempts to represent a diverse population, it may not represent the overall United States population. The results of this study should be interpreted and applied appropriately. In addition, with the cumulative nature of ICD‐10 diagnosis codes in EHR data, as a cross‐sectional study, we could not calculate the number of people having each specific type of dementia. Future longitudinal study designs should be incorporated to assess disease diagnosis and progression. Overall, the findings from this study highlight this crucial initial step towards understanding the heterogeneity of the comorbid condition profiles in persons living with dementia and their care.

## Author Contributions

**Yuchen Zhang:** conceptualization, data curation, formal analysis, investigation, methodology, project administration, resources, software, validation, visualization, writing – original draft, writing – review and editing. **Zhirui Deng:** data curation, formal analysis, methodology, software, supervision, writing – review and editing. **Jennifer Seaman:** conceptualization, investigation, resources, supervision, writing – review and editing. **Theresa A. Koleck:** conceptualization, data curation, formal analysis, investigation, methodology, resources, software, supervision, visualization, writing – review and editing.

## Conflicts of Interest

The authors declare no conflicts of interest.

## Transparency Statement

The lead author Yuchen Zhang affirms that this manuscript is an honest, accurate, and transparent account of the study being reported; that no important aspects of the study have been omitted; and that any discrepancies from the study as planned (and, if relevant, registered) have been explained.

## Supporting information

Supporting information.

## Data Availability

The data that support the findings of this study are available from the All of Us Research Program. Restrictions apply to the availability of these data, which were used under permission and requirements set forth by the All of Us Research Program for this study. Data are available at https://www.researchallofus.org/data-tools/workbench/ with the permission of the All of Us Research Program.

## References

[1] Alzheimer's Association , “2023 Alzheimer's Disease Facts and Figures,” Alzheimer's & Dementia 19, no. 4 (March 2023): 1598–1695. 10.1002/alz.13016 36918389

[2] D. Ma , Y. Wang , Y. Zhao , et al., “How to Manage Comorbidities in People With Dementia: A Scoping Review,” Ageing Research Reviews 88 (July 2023): 101937. 37087058 10.1016/j.arr.2023.101937

[3] M. P. Aranda , I. N. Kremer , L. Hinton , et al., “Impact of Dementia: Health Disparities, Population Trends, Care Interventions, and Economic Costs,” Journal of the American Geriatrics Society 69, no. 7 (July 2021): 1774–1783. 34245588 10.1111/jgs.17345 PMC8608182

[4] H. Bergman , S. Borson , F. Jessen , et al., “Dementia and Comorbidities in Primary Care: A Scoping Review,” BMC Primary Care 24, no. 1 (December 2023): 277. 38097969 10.1186/s12875-023-02229-9 PMC10720181

[5] F. Bunn , A. M. Burn , C. Goodman , et al., “Comorbidity and Dementia: a Scoping Review of the Literature,” BMC Medicine 12, no. 1 (October 2014): 192, 10.1186/s12916-014-0192-4. 25358236 PMC4229610

[6] F. Formiga , I. Fort , M. J. Robles , et al., “Comorbidity and Clinical Features in Elderly Patients With Dementia: Differences According to Dementia Severity,” Journal of Nutrition, Health and Aging 13, no. 5 (May 2009): 423–427.10.1007/s12603-009-0078-x19390748

[7] S. Borson , J. M. Scanlan , M. Lessig , and S. DeMers , “Comorbidity in Aging and Dementia: Scales Differ, and the Difference Matters,” American Journal of Geriatric Psychiatry 18, no. 11 (November 2010): 999–1006. 10.1097/JGP.0b013e3181d695af PMC2962706 20808091

[8] F. Clague , S. W. Mercer , G. McLean , E. Reynish , and B. Guthrie , “Comorbidity and Polypharmacy in People With Dementia: Insights From a Large, Population‐Based Cross‐Sectional Analysis of Primary Care Data,” Age and Ageing 46, no. 1 (January 2017): 33–39. 28181629 10.1093/ageing/afw176

[9] D. Goldfarb , S. Sheard , L. Shaughnessy , and A. Atri , “Disclosure of Alzheimer's Disease and Dementia,” Journal of Clinical Psychiatry 80, no. 2 (March 2019): MS18002BR1C. 10.4088/JCP.MS18002BR1C 30900850

[10] S. K. Kim and M. Park , “Effectiveness of Person‐Centered Care on People With Dementia: A Systematic Review and Meta‐Analysis,” Clinical Interventions in Aging 12, no. 12 (February 2017): 381–397. 28255234 10.2147/CIA.S117637 PMC5322939

[11] F. Gaubert and H. Chainay , “Decision‐Making Competence in Patients With Alzheimer's Disease: A Review of the Literature,” Neuropsychology Review 31, no. 2 (February 2021): 267–287. 33576942 10.1007/s11065-020-09472-2

[12] Y. Zhang , J. H. Lingler , C. M. Bender , and J. B. Seaman , “Dignity in People With Dementia: A Concept Analysis,” Nursing Ethics 31, no. 7 (June 2024): 1220–1232. 38907527 10.1177/09697330241262469

[13] S. L. Kao , J. H. Wang , S. C. Chen , Y. Y. Li , Y. L. Yang , and R. Y. Lo , “Impact of Comorbidity Burden on Cognitive Decline: A Prospective Cohort Study of Older Adults With Dementia,” Dementia and Geriatric Cognitive Disorders 50, no. 1 (March 2021): 43–50. 33789290 10.1159/000514651

[14] S. Sörensen and Y. Conwell , “Issues in Dementia Caregiving: Effects on Mental and Physical Health, Intervention Strategies, and Research Needs,” American Journal of Geriatric Psychiatry 19, no. 6 (June 2011): 491–496. 10.1097/JGP.0b013e31821c0e6e PMC3774150 21502853

[15] E. E. Lee , B. Chang , S. Huege , and J. Hirst , “Complex Clinical Intersection: Palliative Care in Patients With Dementia,” American Journal of Geriatric Psychiatry 26, no. 2 (February 2018): 224–234. 10.1016/j.jagp.2017.06.015 PMC5747550 28822692

[16] National Institutes of Health , All of Us Research Program [Internet], (Nih.gov., 2019), https://allofus.nih.gov/.

[17] K. Bauer , L. Schwarzkopf , E. Graessel , and R. Holle , “A Claims Data‐Based Comparison of Comorbidity in Individuals With and Without Dementia,” BMC Geriatrics 14, no. 1 (January 2014): 10. 24472217 10.1186/1471-2318-14-10 PMC3909381

[18] “Data Snapshots—All of Us Research Hub [Internet] ,” Researchallofus.org, 2024, https://www.researchallofus.org/data-tools/data-snapshots/?_gl=1.

[19] J. A. Hirsch , G. Nicola , G. McGinty , et al., “ICD‐10: History and Context,” American Journal of Neuroradiology 37, no. 4 (January 2016): 596–599. 26822730 10.3174/ajnr.A4696 PMC7960170

[20] M. E. Charlson , D. Carrozzino , J. Guidi , and C. Patierno , “Charlson Comorbidity Index: A Critical Review of Clinimetric Properties,” Psychotherapy and Psychosomatics 91, no. 1 (2022): 8–35. 34991091 10.1159/000521288

[21] W. P. Glasheen , T. Cordier , R. Gumpina , G. Haugh , J. Davis , and A. Renda , “Charlson Comorbidity Index: *ICD‐9* Update and *ICD‐10* Translation,” American Health & Drug Benefits 12, no. 4 (2019): 188–197. 31428236 PMC6684052

[22] M. Hedetoft , M. B. Madsen , L. B. Madsen , and O. Hyldegaard , “Incidence, Comorbidity and Mortality in Patients With Necrotising Soft‐Tissue Infections, 2005–2018: A Danish Nationwide Register‐Based Cohort Study,” BMJ Open 10, no. 10 (October 2020): e041302. 10.1136/bmjopen-2020-041302 PMC7569942 33067303

[23] “Condition Categories—Chronic Conditions Data Warehouse [Internet],” (2013), www2.ccwdata.org, https://www2.ccwdata.org/web/guest/condition-categories.

[24] J. R. Conway , A. Lex , and N. Gehlenborg , “UpSetR: An R Package for the Visualization of Intersecting Sets and Their Properties,” Bioinformatics 33 (2017): 2938–2940. 28645171 10.1093/bioinformatics/btx364 PMC5870712

[25] Public health England , “Dementia: Comorbidities in Patients—Data Briefing [Internet],” GOV.UK, 2019, https://www.gov.uk/government/publications/dementia-comorbidities-in-patients/dementia-comorbidities-in-patients-data-briefing.

[26] S. Sharma , J. Liu , A. C. Abramowitz , et al., “Leveraging Multi‐Site Electronic Health Data for Characterization of Subtypes: A Pilot Study of Dementia in the N3C Clinical Tenant,” JAMIA Open 7, no. 3 (August 2024): ooae076. 39132679 10.1093/jamiaopen/ooae076 PMC11316614

[27] N. Alexander , D. C. Alexander , F. Barkhof , and S. Denaxas , “Identifying and Evaluating Clinical Subtypes of Alzheimer's Disease in Care Electronic Health Records Using Unsupervised Machine Learning,” BMC Medical Informatics and Decision Making 21, no. 1 (December 2021): 343. 34879829 10.1186/s12911-021-01693-6 PMC8653614

[28] X. Y. Ge , K. Cui , L. Liu , et al., “Screening and Predicting Progression From High‐Risk Mild Cognitive Impairment to Alzheimer's Disease,” Scientific Reports 11, no. 1 (September 2021): 17558. 34475445 10.1038/s41598-021-96914-3 PMC8413294

[29] T. H. Edwin , B. H. Strand , K. Persson , K. Engedal , G. Selbæk , and A. B. Knapskog , “Neuropsychiatric Symptoms and Comorbidity: Associations With Dementia Progression Rate in a Memory Clinic Cohort,” International Journal of Geriatric Psychiatry 36, no. 6 (February 2021): 960–969. 33462872 10.1002/gps.5500

[30] V. C. Cavdar , B. Ballica , M. Aric , Z. B. Karaca , E. G. Altunoglu , and F. Akbas , “Exploring Depression, Comorbidities and Quality of Life in Geriatric Patients: A Study Utilizing the Geriatric Depression Scale and WHOQOL‐OLD Questionnaire,” BMC Geriatrics 24, no. 1 (August 2024): 687. 39143531 10.1186/s12877-024-05264-y PMC11325729

[31] “Guiding an Improved Dementia Experience (GUIDE) Model | CMS [Internet],” (2024), www.cms.gov, https://www.cms.gov/priorities/innovation/innovation-models/guide. 10.1016/j.jagp.2025.12.004 PMC12853419 41486046

