# Supplementary material for: Thinking About It All Together: A Descriptive Analysis to Understand Comorbidities in People Living With Dementia
Source: Health Sci Rep. 2025 Feb 5;8(2):e70449. doi: 10.1002/hsr2.70449 (PMC11798731; doi:10.1002/hsr2.70449)
Supplement: Supplementary file 1 — Supporting information. [file HSR2-8-e70449-s001.docx]

**Supplementary Material 1.** ICD-10 diagnosis codes used to define diagnosis of dementia and comorbidities

| ***Condition*** | ***ICD-10 codes*** |
| --- | --- |
| Dementia | F00.x, F01.x, F02.x, F03.x, F05.x, G30.x, G31.x, R41.81 |
| Myocardial Infarction | I21.x, I22.x, I23.x, I25.2 |
| Congestive Heart Failure | I11.0, I13.0, I13.2, I25.2, I42.0x, I43, I50.x, P29.0 |
| Peripheral Vascular Disease | I70.x, I71.x, I73.8, I73.9, I77.1, I79.0, K55.1, Z95.8 |
| Cerebrovascular Disease | G45.x, G46.x, G32.0x, H34.1x, H34.2x, I60.x, I61.x, I62.x, I63.x, I64.x, I65.x, I66.x, I67.x, I68.x |
| Chronic Pulmonary Disease | J40, J41.x, J42, J43.x, J44.x, J45.x, J46.x, J47.x, Z87.01, J60, J61, J62.x, J63.x, J64, J65, J66.x, J67.x, J68.4, J70.1, J70.3 |
| Rheumatologic Disease | M05.x, M06.x, M31.5, M32.x, M33.x, M34.x, M35.1, M35.3, M36.0 |
| Peptic Ulcer | K25.x, K26.x, J27.x, K28.x |
| Hemiplegia | G04.1, G11.4, G80.0, G80.1, G80.2, G81.x, G82.x, G83.x |
| Diabetes | E08.x, E09.x, E10.x, E11.x, E13.x |
| Diabetes Without Complications | E**.0x, E**.1x, E**.6x, E**.8x, E**.9x |
| Diabetes with Chronic Complications | E**.2, E**.3, E**.4, E**.5 |
| Mild Liver Disease | B18.x, K70.x, K71.3, K71.4, K71.5, K71.7, K73.x, K74.x, K76.0, K76.2, K76.3, K76.4, K76.8, K76.9, Z94.4 |
| Severe Liver Disease | I85.0x, I86.4, K70.4x, K71.1x, K72.1x, K72.9, K76.5, K76.6,  K76.7 |
| Mild Renal Disease | I12.9, I13.0, I13.10, N03.x, N05.x, N18.1, N18.2, N18.3, N18.4, N18.9, Z94.0 |
| Severe Renal Disease | I12.0, I13.11, I13.2, N18.5, N18.6, N19, N25.0, Z49.x, Z99.2 |
| Malignancy | C0x.x, C1x.x, C2x.x, C30.x, C31.x, C32.x, C33.x, C34.x, C37.x, C38.x, C39.x, C40.x, C41.x, C43.x, C45.x, C46.x, C47.x, C48.x, C49.x, C50.x, C51.x, C52.x, C53.x, C54.x, C55.x, C56.x, C57.x, C58.x, C60.x, C61.x, C62.x, C63.x, C76.x, C80.1, C81.x, C82.x, C83.x,C84.x, C85.x, C88.x, C9x.x |
| Metastatic Solid Tumor | C77.x, C78.x, C79.x, C80.0 |
| HIV/AIDS | B20, B97.35, R75, Z21 |

Note: the “x” in each ICD-10 code is the wildcard character, which is used as a placeholder to ensure the code meets the required format.
